# Lobocrassin B Induces Apoptosis of Human Lung Cancer and Inhibits Tumor Xenograft Growth

**DOI:** 10.3390/md15120378

**Published:** 2017-12-04

**Authors:** Meng-Xian Lin, Shen-Hao Lin, Yi-Rong Li, Ya-Hsuan Chao, Ching-Hsiung Lin, Jui-Hsin Su, Chi-Chien Lin

**Affiliations:** 1 Institute of Biomedical Science, National Chung-Hsing University, Taichung 40227, Taiwan; g520iloveyou@yahoo.com.tw (M.-X.L.); cmdr.linsh@gmail.com (S.-H.L.); peanutsnoopyemmali@gmail.com (Y.-R.L.); demonsandy@gmail.com (Y.-H.C.); 2 Department of Internal Medicine, Division of Chest Medicine, Changhua Christian Hospital, Changhua 500, Taiwan; teddy@cch.org.tw; 3 National Museum of Marine Biology and Aquarium, Pingtung 944, Taiwan; 4 Department of Health and Nutrition, Asia University, Taichung 413, Taiwan; 5 Department of Medical Research, China Medical University Hospital, Taichung 404, Taiwan

**Keywords:** Lobocrassin B, lung cancer, apoptosis, mitochondria, caspase, xenograft

## Abstract

Lobocrassin B, a natural cembrane-type compound isolated from the soft coral *Lobophytum crassum*, has been shown to have significant biological effects, including anticancer activity. As the most common cause of cancer mortality worldwide, lung cancer remains a major concern threatening human health. In the current study, we conducted in vitro experiments to demonstrate the inhibiting effect of Lobocrassin B on CL1-5 and H520 human lung cancer cells growth and to explore the underlying mechanisms, as well as in nude mice bearing CL1-5 tumor xenografts. Lobocrassin B exerted cytotoxic effects on lung cancer cells, as shown by decreasing cell viability, and inducing apoptosis, oxidative stress and mitochondrial dysfunction. In addition, the increased level of Bax, cleaved caspase-3, -9 and -8, and the suppression of Bcl-2 were observed in the Lobocrassin B treated cells. Moreover, in vivo assays verified the significance of these results, revealing that Lobocrassin B inhibited CL1-5 tumor xenograft growth and that inhibitory effects were accompanied by a marked increase in tumor cell apoptosis. In conclusion, the results suggested that Lobocrassin B could be a potential anticancer compound for its propensity to inhibit growth and induce apoptosis in human lung cancer cells.

## 1. Introduction

Lung cancer is one of the most common causes of cancer-related death worldwide. Siegel et al. estimated the mortality rate of lung cancer-related cases of 2017 in USA could be more than one-quarter (26%) of all cancer deaths [1]. Among these lung cancer cases, the majority of them are non-small cell lung cancer (NSCLC), which has a poor five-year survival rate [2]. Lung cancer is usually detected after development of late stages, and drug resistance often appears later during the course of treatment, leading to unsatisfying outcomes and poor prognosis of patients [3]. Therefore, there is a necessity for the development of novel therapeutic strategies against lung cancer, especially for NSCLC.

Marine natural products have emerged as the new era in discovering therapeutic drugs, including those can against cancer cell [4,5,6]. Lobocrassin B is a natural cembrane-type compound diterpenoid firstly isolated from the soft coral *Lobophytum crassum* in 2011 and has been shown to exhibit a wide variety of biological effects, such as anti-cancer and immunomodulatory activities [7,8]. In specific, Lobocrassin B could not only suppress the activation of immune cells via dampening toll-like receptor (TLR) pathway, but significantly inhibit the product of superoxide anion among other cembrane-type derivatives. Also, it was found to possess moderate cytotoxicity to certain cancer cells, including human leukemia and human hepatoma [7]. However, the molecular mechanisms of this anti-cancer effect by Lobocrassin B remain unknown. In addition, to our knowledge, there has been no studies have been conducted on the anticancer effects of Lobocrassin B against human lung cancer cells.

Thus, in this study, we determined the cytotoxic effects of Lobocrassin B in human CL1-5 and H520 NSCLC cancer cell lines and on xenograft CL1-5 tumor growth in nude mice. We also explore its potential mechanisms underlying the induced cancer cell death.

## 2. Results

### 2.1. Cytotoxic Effects of Lobocrassin B on CL1-5 and H520 Cells

**Figure 1:**
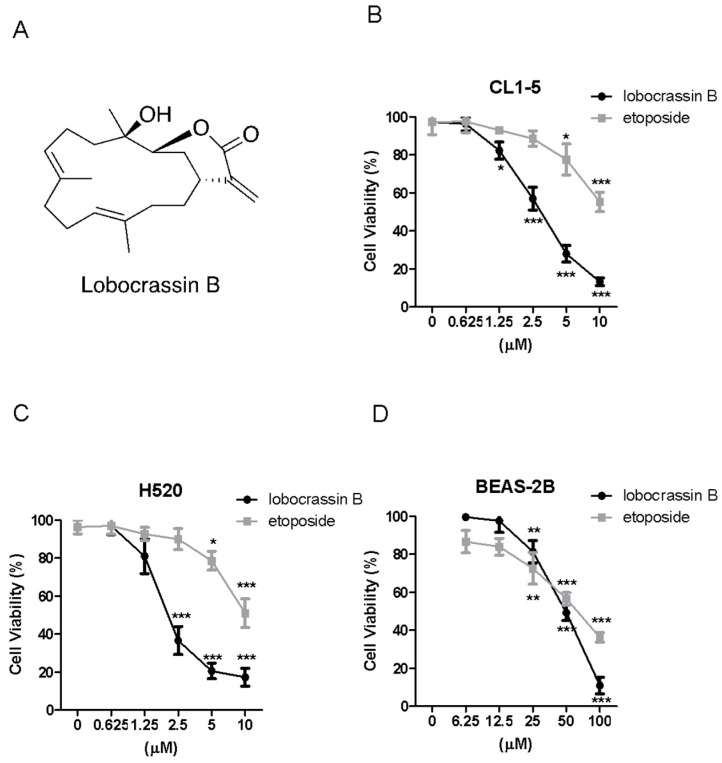
Lobocrassin B exhibit cytotoxicity in CL1-5 and H520 cells. (**A**) Chemical structure of lobocrassin B. The cell viability of (**B**) CL1-5, (**C**) H520 and (**D**) BEAS-2B after treating with Lobocrassin B or etoposide for 24 h was determined by MTT assays. The bar data shown represent the mean ± SD of samples from three wells. * *p* < 0.05; ** *p* < 0.01; *** *p* < 0.001 compared to control group (One-Way ANOVA). Data are representative of at least three independent experiments.

First of all, we investigated whether Lobocrassin B can lead to cell death of two lung cancer cell lines, CL1-5 and H520, as well as a human bronchial epithelial cell line, BEAS-2B, by MTT assay. The results showed that both Lobocrassin B and etoposide as a positive control caused significant cytotoxicity to CL1-5 and H520 cells by 24 h-treatment in a dose-dependent manner (Figure 1B,C), whereas Lobocrassin B exhibited a moderate cytotoxic effect on BEAS-2B cells (Figure 1D). The 50% of inhibitory concentration (IC50) values of Lobocrassin B and etoposide were 4.4 ± 2.3 μM and 11.2 ± 4.3 μM for CL1-5 cells, 3.9 ± 1.7 μM and 10.5 ± 2.3 μM for H520 cells, 53.2 ± 8.6 μM and 66.5 ± 15.6 μM for BEAS-2B cells, respectively, calculated by MTT assay results.

**Figure 2:**
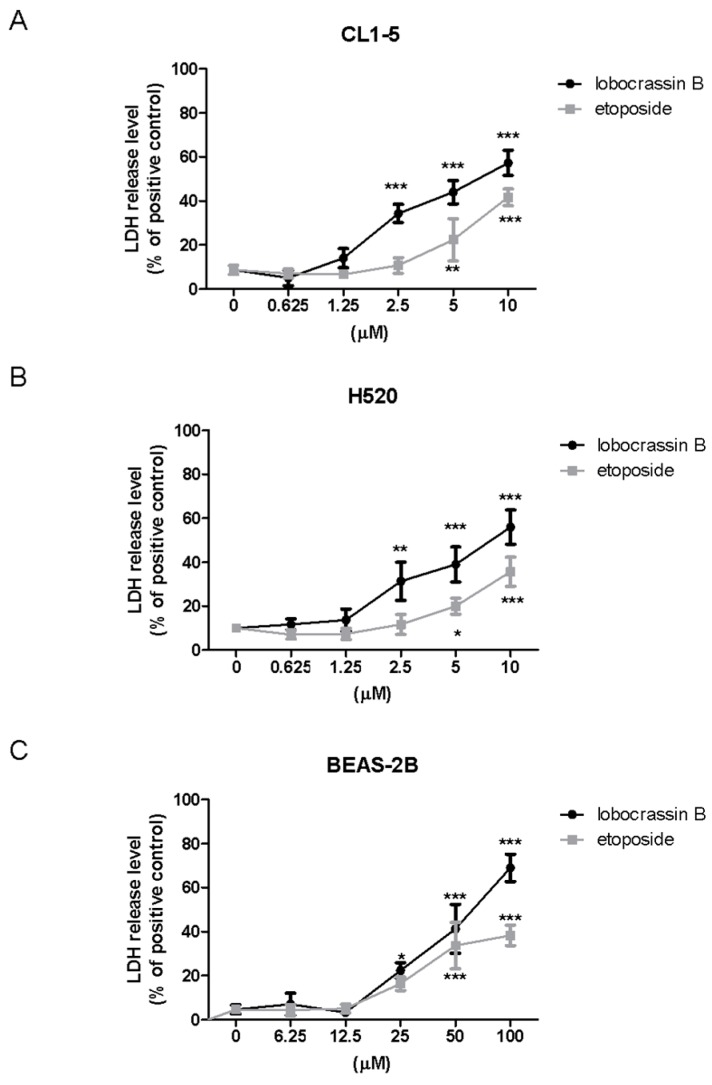
The levels of released lactate dehydrogenase (LDH) in the medium of lobocrassin B-treated cells. (**A**) CL1-5, (**B**) H520 and (**C**) BEAS-2b cells were incubated with lobocrassin B or etoposide for 24 h followed by LDH assays. The bar data shown represent the mean ± SD of samples from three wells. * *p* < 0.05; ** *p* < 0.01; *** *p* < 0.001 compared to control group (One-Way ANOVA). Data are representative of at least three independent experiments.

To further confirm the cytotoxic effects of Lobocrassin B on lung cancer cells, we performed Lactate dehydrogenase (LDH) assay after Lobocrassin B treated CL-15 and H520 cell lines. As shown in Figure 2, Lobocrassin B at 10 µM resulted in 6.2- and 5.4-fold (*p* < 0.01) increases of LDH concentrations in the supernatant of CL1-5 and H520 cells over the untreated control by 24 h of treatment (Figure 2A,B). Notably, while the effective concentrations of Lobocrassin B and etoposide that cause cytotoxicity on two lung cancer cell lines are below 10 µM, the concentrations need to be increased to 25 and 50 µM to observe the significant cytotoxicity in BEAS-2B cells, suggesting that the cytotoxic effect of Lobocrassin B might preferably cause higher cell death in carcinogenic cells than normal cells (Figure 2C).

### 2.2. Lobocrassin B Induces Apoptosis in CL1-5 and H520 Cells

**Figure 3:**
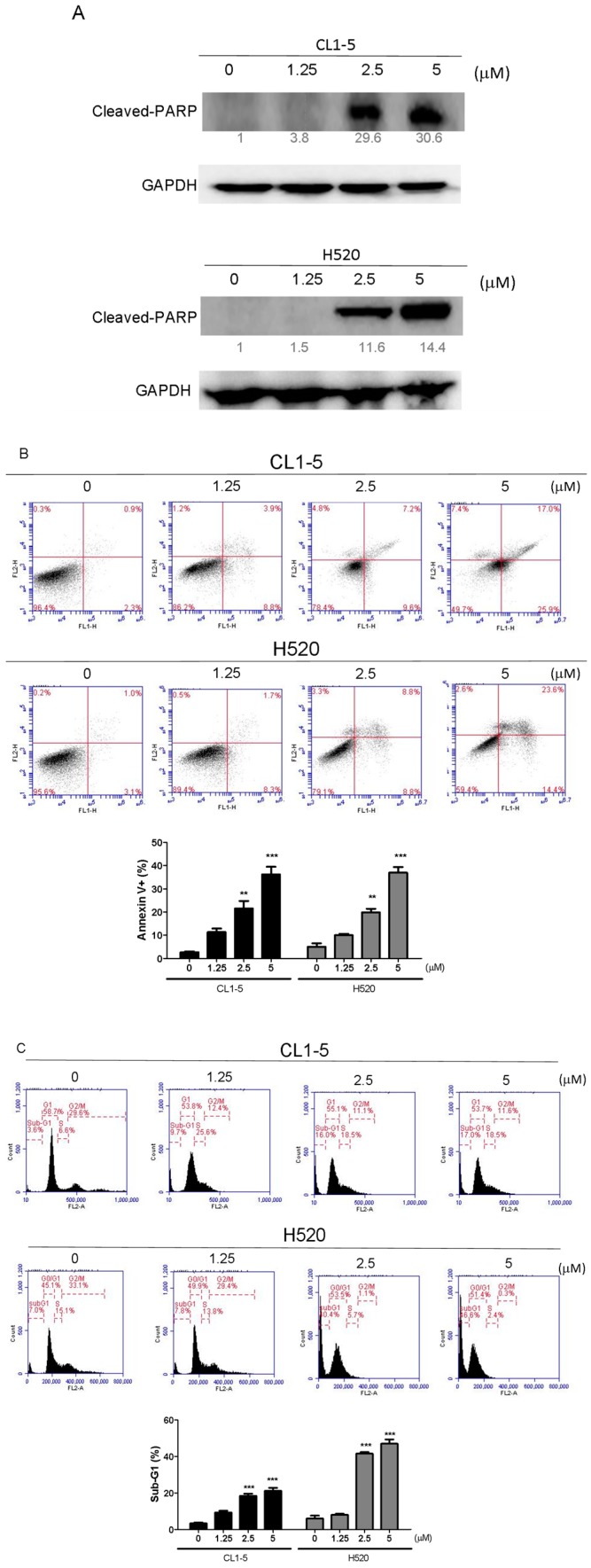
Incubation for 24 h with Lobocrassin B at concentrations of 2.5 and 5 μM strongly enhanced the (**A**) cleaved Poly (ADP-ribose) polymerase (PARP) expression, (**B**) Annexin V positive apoptotic cells, and (**C**) Sub-G1 population levels in CL1-5 and H520 cells. The bar data shown represent the mean ± SD of samples from three wells. ** *p* < 0.01; *** *p* < 0.001 compared to control group (One-Way ANOVA). Data are representative of at least three independent experiments.

Canonically, the activation of Poly (ADP-ribose) polymerase (PARP) canonically serves as a marker for detecting apoptotic cells [9]. By determining the protein expression of cleaved PARP with Western blotting, we found that the Lobocrassin B dose-dependently enhanced the cleavage of PARP in both CL1-5 and H520 cells (Figure 3A). To further clarify the type of that Lobocrassin B used to exert cytotoxicity, apoptosis and cell-cycle distribution were conducted by Annexin V-FITC, and propidium iodide staining and flow cytometry. Both CL1-5 and H520 cells were exposed to a series of concentrations of Lobocrassin B for 24 h. As shown in Figure 2A, the percentage of Annexin V positive cells increased in the CL1-5 and H520 cells, respectively to become apoptotic (Figure 3B). Additionally, the sub-G_1_ population, which is an indication of cell death, increased significantly in the treatment of Lobocrassin B (Figure 3C). These results suggest that Lobocrassin B induces apoptosis in A549 and CL1-5 cells.

### 2.3. Lobocrassin B Treatment Caused Mitochondrial Dysfunction and Reactive Oxygen Species (ROS) Accumulation in CL-15 and H520 Cells

**Figure 4:**
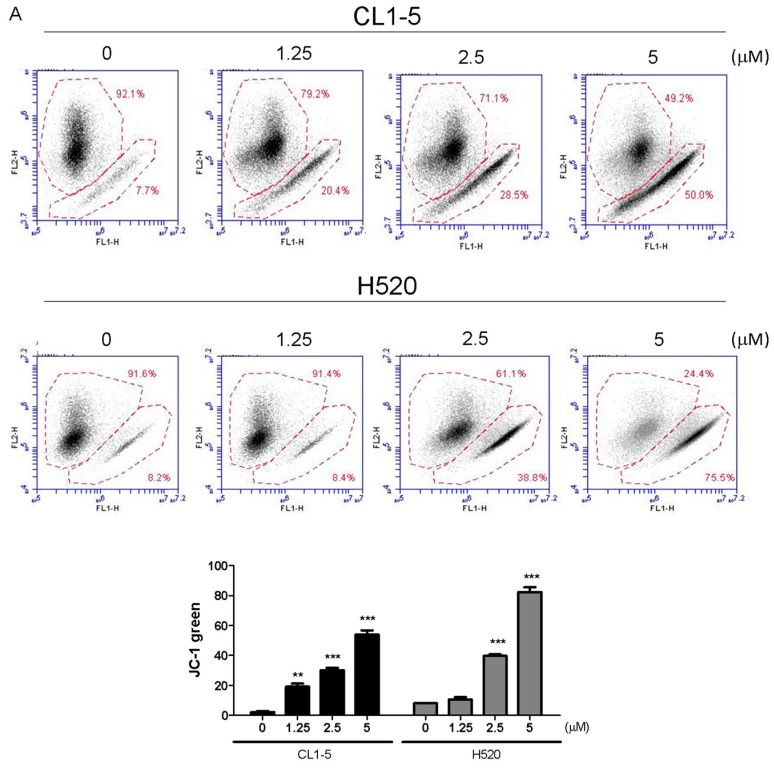

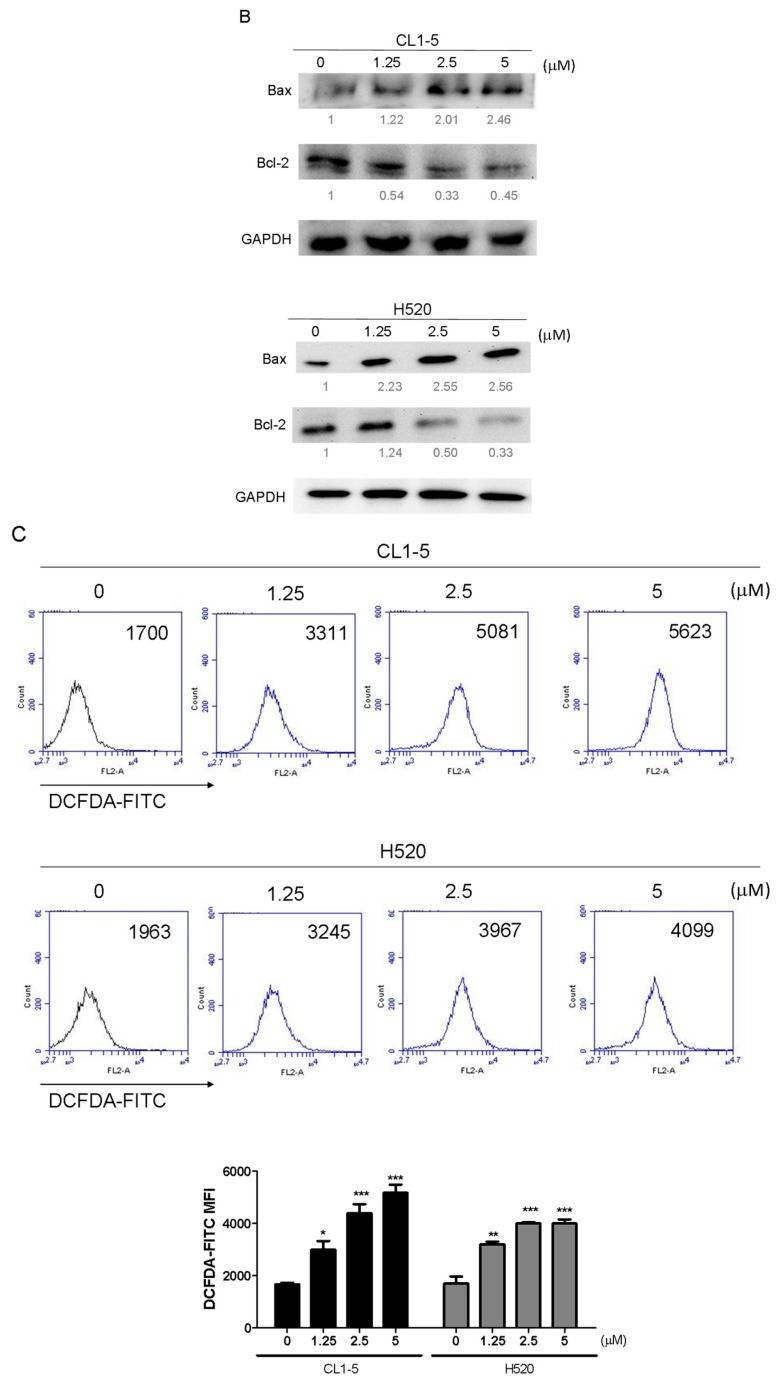
A 24 h incubation with Lobocrassin B at concentrations of 2.5 and 5 μM caused mitochondrial dysfunction and ROS accumulation in CL1-5 and H520 cells. (**A**) The dissipation of ΔΨm was observed detecting by stained with JC-1 dye and analyzed by flow cytometry. (**B**) Lobocrassin B enhanced the levels of Bax and reduced the expression of Bcl-2. The fold of band intensity compared to related controls was marked respectively. (**C**) Lobocrassin B also increased intracellular reactive oxygen species (ROS) levels by stained with DCFH-DA dye and analyzed by flow cytometry. The bar data shown represent the mean ± SD of samples from three wells. * *p* < 0.05; ** *p* < 0.01; *** *p* < 0.001 compared to control group (One-Way ANOVA). All data are representative of at least three independent experiments.

**Figure 5:**
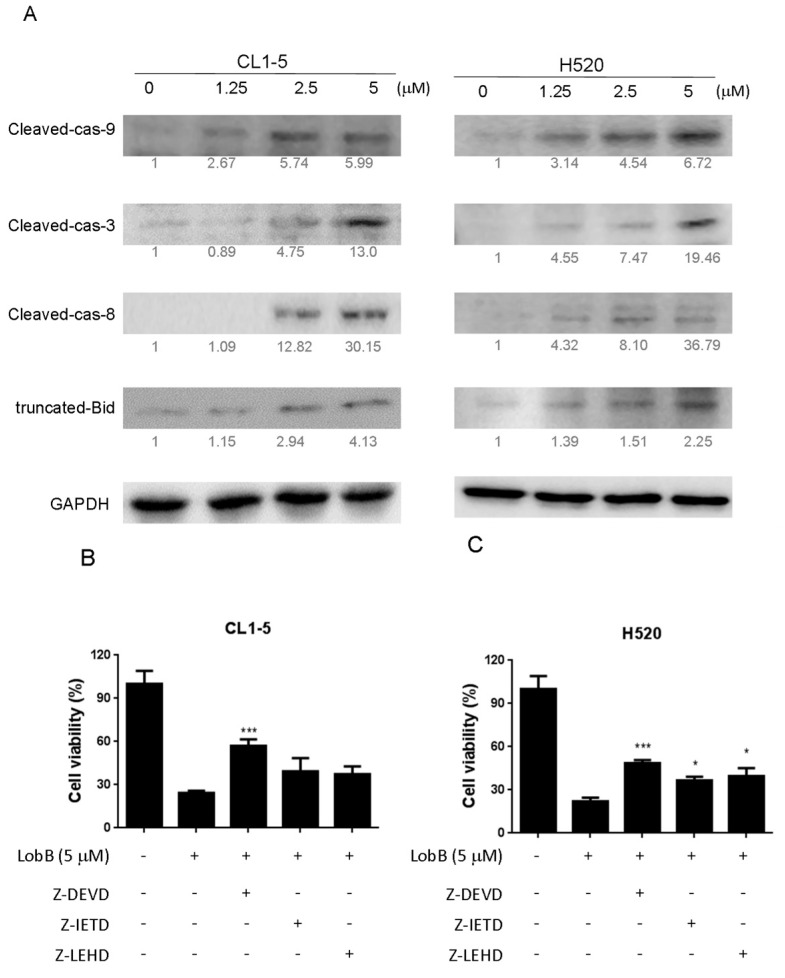
A 24 h incubation with Lobocrassin B at concentrations of 2.5 and 5 μM increased the (**A**) expression of cleaved capsae-9, cleaved capsae-8, cleaved capsae-3, and truncated Bid (tBID) in CL1-5 and H520 cells. The fold of band intensity compared to related controls was marked respectively. When (**B**) CL1-5 and (**C**) H520 cells were preincubated with inhibitors of caspase-3 (50 μM, Z-DEVD-FMK), caspase-8 (50 μM, Z-IETD-FMK), and caspase-9 (50 μM, Z-LEHD-FMK), Lobocrassin B induced cell death was significantly reduced. The bar data shown represent the mean ± SD of samples from three wells. * *p* < 0.05; *** *p* < 0.001 compared to control group (One-Way ANOVA). All data are representative of at least three independent experiments.

Mitochondria play a key role in apoptotic signaling given that link intrinsic and extrinsic apoptotisis pathways at the mitochondria to alter mitochondrial membrane permeabilization (MMP) [10]. To determine whether the mitochondrial apoptotic pathway is involved in Lobocrassin B-induced apoptosis, the effect of Lobocrassin B on MMP (Δψm) was estimated by flow cytometry after staining with JC-1 fluorescent dye. As shown by the decreased JC-1 fluorescence ratio (red/green), Lobocrassin B treatment for 24 h led to reduction in mitochondrial membrane permeabilization (MMP) in the CL1-5 and H520 cells, compared with control cells (Figure 4A). In addition, since MMP during apoptosis is regulated directly by the Bcl-2 family of proteins [11]. Thus, we further examined proapoptotic protein Bax and antiapoptotic protein Bcl-2 expression by western blotting. We found that Lobocrassin B treatment significantly increased the expression level of Bax and decreased of Bcl-2 (Figure 4B). Moreover, it is well documented that mitochondrial dysfunctioning caused by excessive generation of ROS [12]. Therefore, we measured the effects of Lobocrassin B on ROS production by staining with DCFH-DA dye and flow cytometry. Figure 5C data showed that when exposed to Lobocrassin B for 24 h, intracellular ROS levels was significantly increased in the CL1-5 and H520 cells, respectively compared with the controls. All these data suggested that Lobocrassin B exerted cytotoxic effects via accumulation of ROS and the disruption of mitochondrial membrane potential.

### 2.4. Effect of Lobocrassin B on Activation of Caspase Cascade

The death receptor induced activation of the initiator caspases-8 can proteolytically activate Bid, which promotes mitochondrial membrane permeabilization (MMP) and directly activates the executioner caspase-3 [13]. Mitochondrial dysfunction causes the activation of Apaf-1-associated initiator caspases-8 caspase-9, which, subsequently, activates executioner caspase-3 and induces the cleavage of PARP [14]. Therefore, in order to understand mechanism of cell death caused by Lobocrassin B CL-15 and H520 cells were treated with different concentration of Lobocrassin B and expression of caspases-8, tBId and caspase-9, caspase-3 were analyzed by immunoblotting analysis. As shown in Figure 5, in Lobocrassin B treatment on CL1-5 and H520 cells for 24 h resulted in increased the cleaved caspase-8, tBid, cleaved caspase-9 and downstream cleaved caspase-3 expression (Figure 5A). Moreover, in the presence of caspase-3 inhibitor Z-DEVD-FMK, caspase-8 inhibitor Z-IETD-FMK, caspase-9 inhibitor Z-LEHD-FMK significantly increased the viability of the cells treated with Lobocrassin B. Meanwhile, the caspase-3 inhibitor showed significant inhibitory effect on Lobocrassin B induced reduction in cell viability (Figure 5B,C) and the caspase-8 and caspase-9 inhibitors partially blocked Lobocrassin B -induced reduction in cell viability (Figure 5B,C). These data suggesting that both the coordination of caspase-8 (extrinsic) and caspase-9 (intrinsic) mediated apoptotic pathway were involved in the Lobocrassin B-induced apoptosis.

### 2.5. Lobocrassin B Inhibits the Growth of CL1-5 Tumor Xenografts

**Figure 6:**
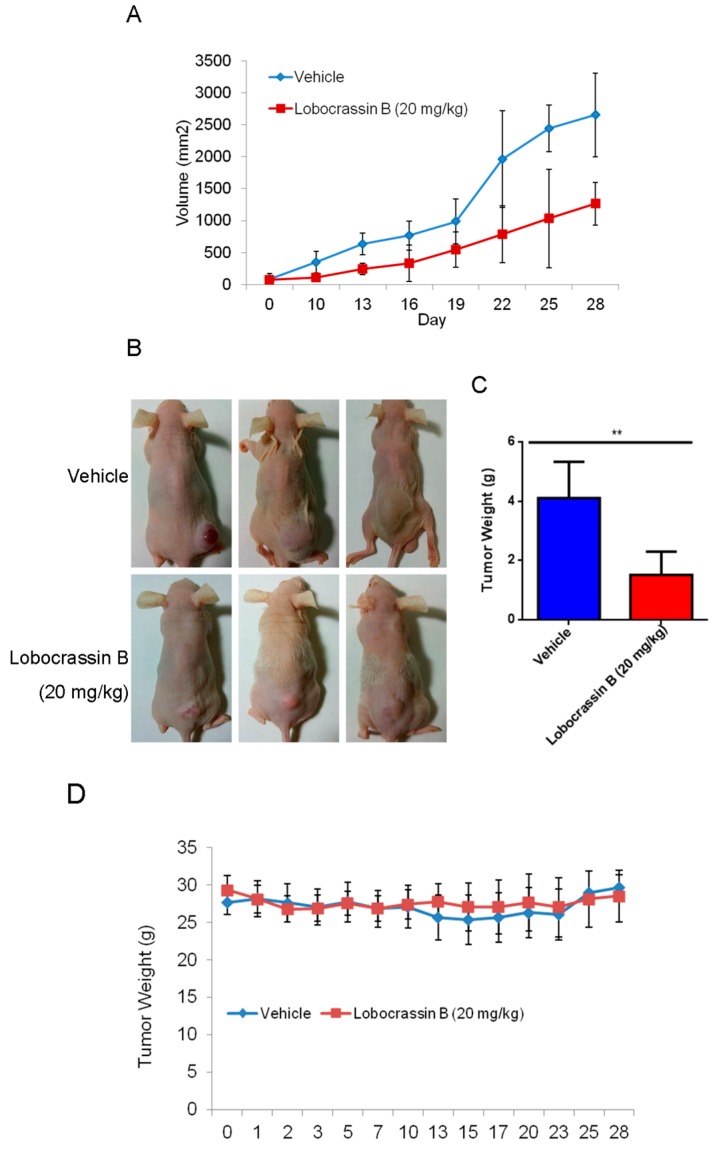

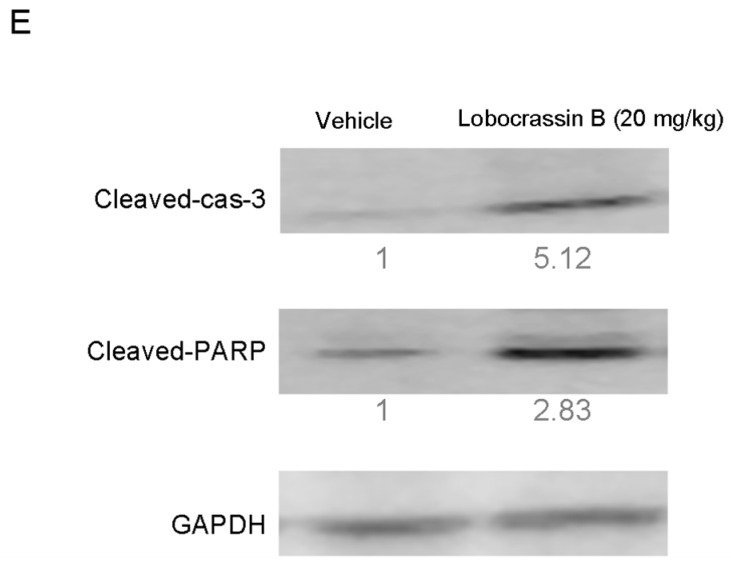
Lobocrassin B inhibits the growth of CL1-5 tumor xenografts in nude mice. Nude mice bearing CL1-5 tumors were treated for 28 days. (**A**) Growth curves of CL1-5 tumor xenografts in Lobocrassin B-treated and untreated mice. Tumor sizes were measured every three days. Data are expressed as the means ± SD (*n* = 5). * *p* < 0.05 were the comparisons between vehicle and Lobocrassin B groups (two-way ANOVA). (**B**) Photograph of tumor tissue (**C**) and the tumor weight at day 28 were examined. ** *p* < 0.01 were the comparisons between vehicle and Lobocrassin B groups (student *t*-test). (**D**) Body weight of Lobocrassin B-treated and untreated mice (**E**) Lobocrassin B reduced the cleaved caspase-3 and caspase-8 expression in tumor tissues from lobocrassin B treated mice. The fold of band intensity compared with the vehicle treated mice was marked respectively. All data are representative of at two independent experiments.

The inhibitory effect of Lobocrassin B on human lung cancer cell proliferation in vitro suggested that it might suppress tumor growth in vivo. To verify this hypothesis, nude mice were transplanted s.c. with CL1-5 cells, followed by 20 mg/kg Lobocrassin B treatment on day 10 after tumor injection. As shown in Figure 6A,B, Lobocrassin B administration significantly suppressed the growth of CL1-5 tumor in nude mice (*p* < 0.05). In addition, compared with the vehicle controls, Lobocrassin B significantly decreased the tumor weight on the 28 (*p* < 0.01; Figure 6C). The Lobocrassin B did not to influence the body weight of the mice compared with the vehicle treatment, which suggested limited side-effects (Figure 6D). Additionally, in the tumor tissues, Lobocrassin B treatment caused a significantly increased the cleaved caspase-3 and PARP expression compared with vehicle treatment (Figure 6E). These results suggested that Lobocrassin B also inhibits the lung cancer cells growth and induces apoptosis in vivo.

## 3. Discussion

In the present study, the cytotoxic effects of Lobocrassin B on human lung cancer were confirmed in CL1-5 and H520 cells and tumor xenograft on athymic nude mice. The Lobocrassin B–induced cell death on the human lung cancer cell lines, as evidenced by a decrease in cell viability, and an increase levels of ROS, as well as the activation of caspase-8 and induction of mitochondrial apoptotic pathway, revealed the possible mechanisms involved.

Mitochondrial functions play central roles in activating apoptosis in mammalian cells. The functional impairment of mitochondria is associated to the loss of MMP [15], which was noted in this study following treatment with the Lobocrassin B for 24 h. The increased apoptotic Bax expression and decreased anti-apoptotic Bcl-2 expression were also observed in the Lobocrassin B -treated cells. The Bcl-2 family is made up of outer mitochondrial membrane proteins that can control mitochondrial-mediated apoptosis [16]. Furthermore, in this study, the increased of intracellular ROS level was detected in the cells treated with the Lobocrassin B. The increased of ROS causes oxidative stress, which in turn can induce mitochondrial apoptosis and cellular dysfunction [10]. It has been reported that ROS challenge can cause mitochondrial permeability transition pore (mPTP) opening, which leads to mitochondrial inner membrane permeabilization, membrane potential dissipation and Cyto c release [17]. Taken together, the effects of Lobocrassin B on mitochondrial function are involved in its anti-lung cancer effects.

On the other hand, apoptosis can be induced through two main apoptotic pathways: the extrinsic (death receptor pathway) and the intrinsic (mitochondrial pathway). Caspase-3 is considered to be the most important of the executioner caspase that can be activated by a mitochondrial pathway involving initiator caspase-9 or a death receptor pathway involving initiator caspase-8 [18]. Activation of caspase-3 activation leads to the cleavage of number important substrates, including PARP. Since caspase-8 cleaves Bcl-2 family protein, Bid into tBid cause mitochondria membrane potential disruption and then also lead to trigger activation of caspase-9, a cross-talk exists between the extrinsic and intrinsic apoptotic pathways [19]. In the present study, we found the cleavage of PARP and the activation of caspase-3 were induced after lobocrassin B treatment, and caspase-3 inhibitor effectively prevented lobocrassin B—induced cell viability reduction. These dates suggest that apoptosis induced by lobocrassin B may be caspase-dependent. At the same time, we also found the activation of initiator caspase-8 and -9 in CL1-5 and H520 cells after lobocrassin B treatment. Furthermore, either Z-IETD-FMK (caspase-8 inhibitor) or Z-LEHD-FMK (caspase-9 inhibitor) only partially reverses lobocrassin B -induced reduction in cell viability in CL1-5 and H520 cells. Therefore, we suggest that combine activation of extrinsic (death receptor pathway) and the intrinsic (mitochondrial pathway) are involved in lobocrassin B -induced apoptosis in human lung cancer cells.

In conclusion, our study showed for the first time that Lobocrassin B exerts an anti–lung cancer effect in cell lines and in tumor xenograft in nude mice. Lobocrassin B treatment increases the ROS generation, MMP dissipation, and caused caspase cascade. These findings reveal that Lobocrassin B could efficiently induce lung cancer death through mitochondrial-dependent apoptotic pathway and it may be a potential chemotherapeutic agent.

## 4. Material and Methods

### 4.1. Cell Culture

The CL1-5 lung adenocarcinoma cell line was provided by Dr. Jeremy J. W. Chen (University of National Chung Hsing University, Taichung City, Taiwan). The lung squamous cell carcinoma cell line H520 and the immortalized bronchial epithelial cell line BEAS-2B were purchased from Food Industry Research and Development Institute (Hsinchu City, Taiwan). All cell lines were maintained in Dulbecco’s modified Eagle’s medium (DMEM), supplemented with a 10% heat inactive fetal bovine serum (FBS), 100 U/mL penicillin and 100 g/mL streptomycin. Cells were incubated at 37 °C in a humidified atmosphere containing 5%/95% of CO_2_/air. Cell culture reagents were purchased from Invitrogen Life Technologies (Carlsbad, CA, USA).

### 4.2. Chemicals

Lobocrassin B was isolated from the wild-type soft coral *Lobophytum crassum* as previously described [8]. Etoposide was purchased from Sigma-Aldrich (Ann Arbor, MI, USA) as a positive control for cell viability assays. Both of Lobocrassin B and etoposide were dissolved in dimethyl sulfoxide (DMSO; Sigma-Aldrich, St. Louis, MO, USA) at 100 mM as a stock solution prior to performing further assays.

### 4.3. MTT Cell Viability Assay

The cells were seeded into 24-well plates at 2 × 10^4^ cells/well and treated with increasing concentrations of Lobocrassin B or etoposide or DMSO (0.1%) as vehicle control for 24. Then, 10 μL of 3-(4,5-dimethylthiazol-2-yl)-2,5-di-phenyltetrazolium bromide (MTT, Sigma-Aldrich, St. Louis, MO, USA) solution (0.5 mg/mL final concentration) was added to each well. Following a 4-h reaction, the supernatant was aspirated, and then 600 μL DMSO were added. The absorbance was examined at 540 nm using a microplate reader (TECAN, Durham, NC, USA). Data were showed as a percentage of absorbance of Lobocrassin B treated cells relative to DMSO-treated cells. The IC50 values are calculated using SPSS 16.0 software (IBM Corporation, Armonk, NY, USA).

### 4.4. Lactate Dehydrogenase (LDH) Assay

LDH cytotoxicity assay kit (Cayman Chemical Co., Ann Arbor, MI, USA) was employed to assess the cellular toxicity induced by compounds. Lung cells were treated with different concentrations of Lobocrassin B or etoposide for 24 h followed by incubating with Triton X-100, and centrifuging at 1200 rpm for 5 min to obtain the supernatant. 100 µL/well of the supernatant was transferred to a new 96-well plate and equal volume of LDH reagent was added to each well. After incubating for 30 min at room temperature, the concentration of LDH was measured with a microplate reader (TECAN, Durham, NC, USA) at wavelength of 490 nm. The LDH release level (% of positive control) was recorded as the percentage of (OD_test_ − OD_blank_)/(OD_positive_ − OD_blank_), where OD_test_ is the optical density of cells treated with either 0.1% DMSO or Lobocrassin B; OD_positive_ is the optical density of etoposide-treated cells and OD_blank_ is the background optical density of the wells without cells.

### 4.5. Measurement of Apoptosis by Annexin V-FITC/PI Staining

The cells (2 × 10^5^/well) were seeded into 6-well plates and treated with 1.25 and 2.5, 5 μM Lobocrassin B for 24 h and then harvested and washed with phosphate buffered saline (PBS). Staining went along with with 5 μL Annexin V-FITC (20 μg/mL) and 5 μL propidium iodide (PI; 50 μg/mL) (BD Biosciences, Franklin Lakes, NJ, USA) in the dark at room temperature for 15 min. The apoptotic cells were measured using an AccuriTM C5 cytometer (BD Biosciences, San Jose, CA, USA).

### 4.6. Caspase Inhibitor Assay

The cells were seeded into 24-well plates at 2 × 10^4^ cells/well and were grown overnight and then pre-treated with inhibitors of caspase-3 (Z-DEVD-FMK, 50 µM, BioVision, San Francisco, CA, USA), caspase-8 (Z-IETD-FMK; 50 µM, San Francisco, CA, USA), and caspase-9 (Z-LEHD-FMK; 50 µM, San Francisco, CA, USA) for 2 h. Cells were then treated with 5 μM Lobocrassin B for 24 h. The cell viability were examined by MTT assay.

### 4.7. DNA Content by Flow Cytometric Analysis

Cells (2 × 10^5^/well) were subcultured into 6-well plates and treated with 1.25 and 2.5, 5 μM Lobocrassin B for 24 h. After challenge, cells were harvested by trypsinization and washed with PBS, fixed in 70% (*v*/*v*) ethanol at −20 °C overnight. Afterward, cells were stained went along with 500 μL PBS with 0.1% (*v*/*v*) Triton X-100, 100 μg/mL RNase A and 50 μg/mL propidium iodide (PI) (Sigma-Aldrich, St. Louis, MO, USA) in constant darkness at room temperature for 20 min. Sub-G1 population containing apoptotic cells was analyzed using an AccuriTM C5 cytometer.

### 4.8. Detection of Reactive Oxygen Species (ROS) and Mitochondrial Membrane Potential (MMP) 

Cells (2 × 10^5^/well) were subcultured into 6-well plates and treated with 1.25 and 2.5, 5 μM Lobocrassin B for 12 h. Thereafter, cells were suspended and incubated in 500 μL of dichlorodihydrofluorescein diace-tate (DCFH-DA, Molecular probes, Eugene, OR, USA) (10 μM) for ROS examination and 2 μM 5,5′,6,6′-tetra chloro-1,1′,3,3′-tetraethylbenzimidazolylcarbocyanine iodide (JC-1; Sigma-Aldrich, St. Louis, MO, USA) (10 µg/mL) for mitochondrial membrane potential in constant darkness at room temperature for 30 min. The samples were analyzed using a using an AccuriTM C5 cytometer.

### 4.9. Western Blot Analysis

Cells (2 × 10^5^/well) were seeded into 6-well plates and treated with indicated concentrations of Lobocrassin B for 24 h. The cells and collected tumor tissues were lysed by RIPA buffer (Sigma-Aldrich, St. Louis, MO, USA) containing 1% protease inhibitor cocktail (Sigma-Aldrich, St. Louis, MO, USA) and 2% phenylmethanesulfonyl fluoride (PMSF; Sigma-Aldrich, St. Louis, MO, USA). Equal loading was verified by using the BCA Protein Assay Kit (Thermo Fisher Scientific, Waltham, MA, USA), to examine protein concentration. The samples of cell lysates were separated by 12% SDS-PAGE gel, and then electroblotted onto Immobilon-P Transfer Membrane (Merck Millipore, Billerica, MA, USA).The membranes were hybridized with anti-Bcl-2 (ab32124), anti-Bax (ab7977), anti-cleaved caspase-3 (ab2302), anti- caspase-8 (ab220171), anti-cleaved caspase-9 (ab2324), anti-cleaved PARP (ab32064) and glyceralde-hyde 3-phosphate dehydrogenase (GAPDH) (ab8245) (Abcam, Cambridge, MA, USA) antibodies at 4 °C overnight after being blocked with PBS containing 0.1% (*v*/*v*) Tween 20 and 5% (*w*/*v*) nonfat dry milk for 3 h at room temperature. The membranes were then incubated with appropriate horseradish peroxidase (HRP)-conjugated secondary antibodies (Jackson ImmunoResearch Laboratories, West Grove, PA, USA) at 4 °C overnight. Band detection was visualized utilizing the enhanced chemiluminescence detection kit reagent (GE Healthcare Life Sciences, Piscataway, NJ, USA). All bands in the blots were normalized to the level of GAPDH for each lane. The intensity of the bands was quantified using ImageJ 1.47 program for Windows from the National Institute of Health (NIH) (Bethesda, MD, USA).

### 4.10. Animals’ Experimentation

Sex-week-old female BALB/c athymic nude mice were purchased from the National Laboratory Animal Center (Taipei, Taiwan). The all animal experiments were approved by the Institutional Animal Care and Use Committee (IACUC) of National Chung Hsing University (Taichung, Taiwan). The mice were housed at a constant room temperature and maintained on a 12 h light/dark cycle with fed a standard rodent diet and water.

### 4.11. Tumor Xenograft Model

CL1-5 cells (1 × 10^7^/mouse) in 0.2 mL at 1:1 mixture of cultural medium and BD Matrigel Basement Membrane Matrix (BD Biosciences, Bedford, MA, USA) was inoculated subcutaneously into the flank of nude mice. When the tumor reached approximately 2 mm^3^ (at day 10 after cell inoculation), the mice were randomly divided into two groups (*n* = 6 each), and intraperitoneal (i.p.) injection included 20mg/kg Lobocrassin B or vehicle (10% DMSO + 90% glyceryl trioctanoate) (Sigma-Aldrich, St. Louis, MO, USA) every other day continuously for three weeks. During the Lobocrassin B administration, the body weight and tumor sizes (mm^3^) were recorded. The formula of length × (width)^2^ × 0.5 was applied to calculate the tumor size. At the end of treatment, all the mice were sacrificed after 28 days. The tumor tissues were measured weighted after being removed. The tumors were dissected and evaluated by immunoblotting as mentioned earlier.

### 4.12. Statistical Analysis

The results are expressed as the means ± standard deviation (SD) in triplicate. For comparison between two groups, we used unpaired two-tailed *t* test (Student’s *t* test). One-way or two-way ANOVA was used to compare multiple groups according to the experiments (GraphPad Prism Version 5.0 (San Diego, CA, USA). The level of statistical significance was considered as a probability (*p*) value b 0.05.
